# PlasmaBlade versus Electrocautery for Deep Inferior Epigastric Perforator Flap Harvesting in Autologous Breast Reconstruction: A Comparative Clinical Outcome Study

**DOI:** 10.3390/jcm13082388

**Published:** 2024-04-19

**Authors:** Angela Augustin, Ines Schoberleitner, Sophie-Marie Unterhumer, Johanna Krapf, Thomas Bauer, Dolores Wolfram

**Affiliations:** Department of Plastic, Reconstructive and Aesthetic Surgery, Medical University of Innsbruck, 6020 Innsbruck, Austria

**Keywords:** autologous breast reconstruction, clinical outcome, electrosurgery, flap harvesting, PEAK PlasmaBlade

## Abstract

(1) **Background**: DIEP-based breast reconstruction necessitates wide undermining at the abdominal donor site, creating large wound areas. Flap harvesting is usually conducted using electrosurgical dissection devices. This study sought to compare the clinical outcomes in patients after using the PEAK PlasmaBlade (PPB) versus monopolar electrocautery (MPE). (2) **Methods**: This retrospective cohort study included 128 patients with DIEP-based breast reconstruction. Patient characteristics and information on the postoperative course were collected and a comparative evaluation was conducted. (3) **Results**: The MPE group exhibited significantly (p* = 0.0324) higher abdominal drainage volume (351.11 ± 185.96 mL) compared to the PPB group (279.38 ± 183.38 mL). A subgroup analysis demonstrated that PPB significantly reduced postoperative wound fluid in patients with BMI > 30 kg/m^2^ (p* = 0.0284), without prior neoadjuvant chemotherapy (p** = 0.0041), and among non-smokers (*p* = 0.0046). Furthermore, postoperative pain was significantly (p**** < 0.0001) lower in the PPB cohort. (4) **Conclusions**: This study confirms the non-inferiority of the PEAK PlasmaBlade to conventional electrocautery for abdominal flap harvesting. The PPB demonstrated advantages, notably reduced drainage volume and lower postoperative pain levels. Recognizing patient subsets that benefit more from the PPB highlights the importance of personalized device selection based on patient characteristics.

## 1. Introduction

Abdominal-based flaps, particularly the deep inferior epigastric perforator (DIEP) flap, have become the standard approach for autologous breast reconstruction following nipple-sparing mastectomy (NSME) or skin-sparing mastectomy (SSME) procedures [1,2,3]. This technique offers several notable advantages, including an enhanced quality of life and greater patient satisfaction when compared to implant-based approaches [4,5,6]. However, the trade-offs are longer surgical duration, extended hospital stay and the necessity to sacrifice an additional donor site. Considering this additional effort, our objective should be to minimize the risks associated with the additional surgery for the patients and continuously strive to enhance the surgical outcome.

Abdominal flap harvesting is performed with electrosurgical devices, using high-frequency electrical current for tissue dissection and simultaneous hemostasis [7]. Different modalities are available, the conventional monopolar electrocautery (MPE) uses a continuous waveform of radiofrequency energy via an uninsulated metal electrode for tissue cutting through thermal ablation, operating at temperatures between 180 and 240 °C [8]. In contrast, the PEAK PlasmaBlade (PPB) employs short (40 µs) high-frequency pulses of radiofrequency energy to generate electrical plasma along an insulated electrode’s edge and it maintains a lower operating temperature around 45 °C [8,9]. Previous investigations suggest that the PlasmaBlade may offer advantages over electrocautery, demonstrating reduced thermal injury depth and inflammatory responses [8,10,11]. Inflammatory processes and trauma to the lymphatic network during surgical dissection are known factors contributing to postoperative seroma formation, a common complication after DIEP flap harvesting, with reported incidences ranging from 1.4% to 16.2% [12,13,14,15,16]. Prolonged drainage duration to prevent seroma formation poses disadvantages such as extended hospitalization and the risk of ascending infections through the drain tube. Previous research has generated conflicting and inconclusive findings regarding whether the choice of the surgical dissection device significantly impacts clinical outcomes. While some studies suggest benefits associated with using the PPB, such as reduced seroma rates and shorter drain dwelling times, these studies have limitations, notably small sample sizes and none have evaluated patient-specific risk factors in this context [11,17,18,19,20,21,22].

Our study seeks to evaluate the influence of the dissection device utilized during abdominal flap harvesting on clinical outcomes, while considering risk factors, such as body mass index (BMI), smoking status and previous chemotherapy.

## 2. Materials and Methods

This retrospective, single-center cohort study was approved by the Institutional Ethics Committee of Medical University Innsbruck (protocol code 1082/2021).

### 2.1. Patients

We included a total of 128 patients who underwent autologous DIEP flap breast reconstruction at our department between 2013 and 2020. Among these, 56 patients underwent abdominal flap harvesting using the PEAK PlasmaBlade (Medtronic, Dublin, Ireland) (PPB), while 72 patients underwent the procedure using monopolar electrocautery (MPE). Assignment to the surgical device was carried out chronologically. Initially, flap dissection with monopolar electrocautery was the standard procedure until the PEAK PlasmaBlade became consistently available at our department. Subsequently, the PPB replaced monopolar electrocautery as the standard dissection device. Inclusion criteria were defined as age over 18 years, uni- or bilateral NSME or SSME, and immediate or staged DIEP-based autologous breast reconstruction by or under the supervision of one single surgeon, who has been a senior member and specialized microsurgeon for over 10 years by the time of the first included patient in this study. To prevent bias, one patient was excluded due to the development of extreme seroma formation (1730 mL). In the case of the excluded patient, flap dissection was performed using monopolar electrocautery. This patient was a 73-year-old undergoing unilateral primary breast reconstruction with a BMI of 30.3 kg/m^2^, non-smoker and without prior chemotherapy. Serous-sanguineous abdominal drainage fluid was highly elevated during the first 48 postoperative hours leading to revision under general anesthesia due to postoperative bleeding and a hematoma on the second postoperative day.

**Figure 1:**
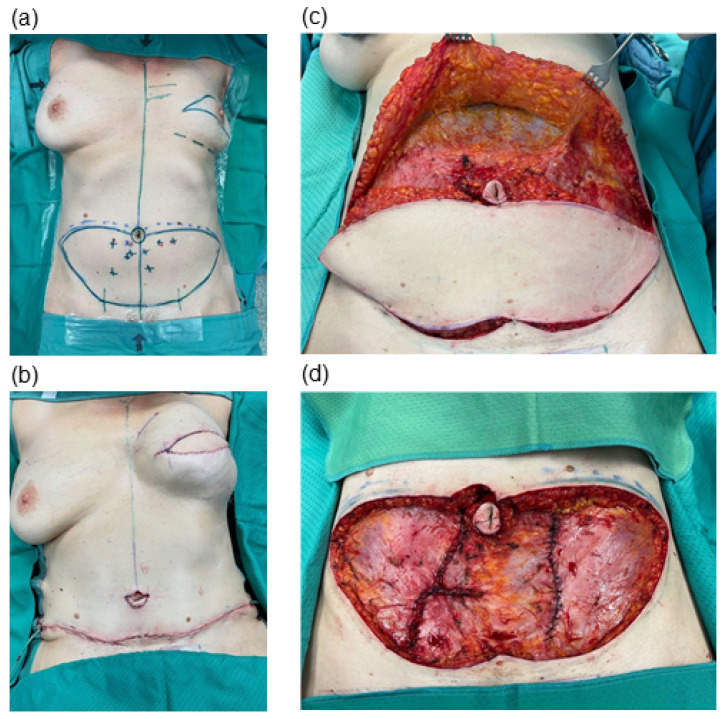
Intraoperative photo documentation of abdominal DIEP flap harvesting. (**a**) Preoperative markings of planned incisions. (**b**) Reconstructive result after skin-sparing mastectomy (resection weight: 650 g) on the left patient side and primary reconstruction (flap weight: 720 g). (**c**) Wound bed after mobilization, with the DIEP flap still in situ. (**d**) Defect after completion of DIEP flap harvesting (resection weight: 750 g).

We conducted a retrospective chart analysis to assess patient demographics, complications and clinical outcomes. All patients included in this study underwent abdominal flap harvesting and received postoperative care following our institutional protocol. We made skin incisions using a steel scalpel and proceeded with subcutaneous tissue preparation using the electrosurgical device. Blood vessels were coagulated using isolated forceps and cautery. To ensure safe perforator identification and dissection, we did not leave fatty tissue on the central aspects of the fascia abdominalis within the flap harvesting area. Perforator vessels were dissected using scissors and bipolar cauterization or surgical clips for hemostasis. Following flap harvest, we mobilized the tissue above the umbilicus in the central area using the electrosurgical device until closure with mild to moderate tension was feasible, if necessary, extending the mobilization to the sub-xiphoid area. During this final step, a fatty layer was left over the fascia to prevent seroma formation. However, we did not mobilize the lateral abdomen to optimize perfusion of the remaining abdominal cutaneous and subcutaneous tissue. Redon drains were placed at the end of surgery, just before wound closure, and were placed under suction (Figure 1). Drain removal was undertaken when the output was less than 30 mL per 24 h. The volume of drainage fluid was recorded at 24 h intervals. All patients were required to wear an abdominal binder for a duration of six weeks following surgery.

For the PEAK PlasmaBlade, surgeries were conducted using standardized technical settings, with both the “cut” and “coagulate” functions set to intensity “6”. These settings remained consistent across all patients, without individual adjustments. In the monopolar electrocautery group preferred settings were typically “cut” 40 W and “coagulation” 40 W. However, minor variations cannot be excluded due to incomplete documentation.

Postoperative pain levels were assessed at various time intervals within a 24 h period using the Numerical Analog Scale, which utilizes a scale ranging from 0 (indicating the absence of pain) to 10 (representing the most severe imaginable pain). If multiple values were recorded per day, the maximum daily score was included in the study.

### 2.2. Statistical Analysis

Statistical analysis was performed using Prism Software 10 for macOS (GraphPad Software, Boston, MA, USA) and Google Sheets (Google LLC, Mountain View, CA, USA; https://www.google.com/sheets/about/, accessed on 30 October 2023). To assess significant differences between the two groups, we employed the independent sample Student’s *t*-test for continuous data. For categorical data, Fisher’s exact and Chi-Square tests were utilized to determine statistical significance. A two-way analysis of variance (ANOVA) test was conducted to assess interactions between the types of surgical devices used (PPB versus MPE) and their individual effects on postoperative pain. Statistical significance of simple linear regression has been determined by comparison of slopes and intercepts with a confidence interval of 95%. A *p*-value of <0.05 was considered significant. The significance threshold was set at *p* < 0.05. The level for statistical significance was set at *p*^ns^ ≥ 0.05, p* < 0.05, p** < 0.02, p*** < 0.001 and p**** < 0.0001 for all statistical tests.

## 3. Results

All 128 patients included in the study were categorized into two distinct cohorts based on the method employed for abdominal flap preparation: either utilizing the PEAK PlasmaBlade (PPB; *n* = 56) or the monopolar electrocautery (MPE; *n* = 72). An analysis of patient characteristics revealed no statistically significant distinctions between the two cohorts in terms of age, BMI, smoking status, neoadjuvant oncologic therapy, diabetes mellitus and the indication for mastectomy (Table 1).

**Figure 2:**
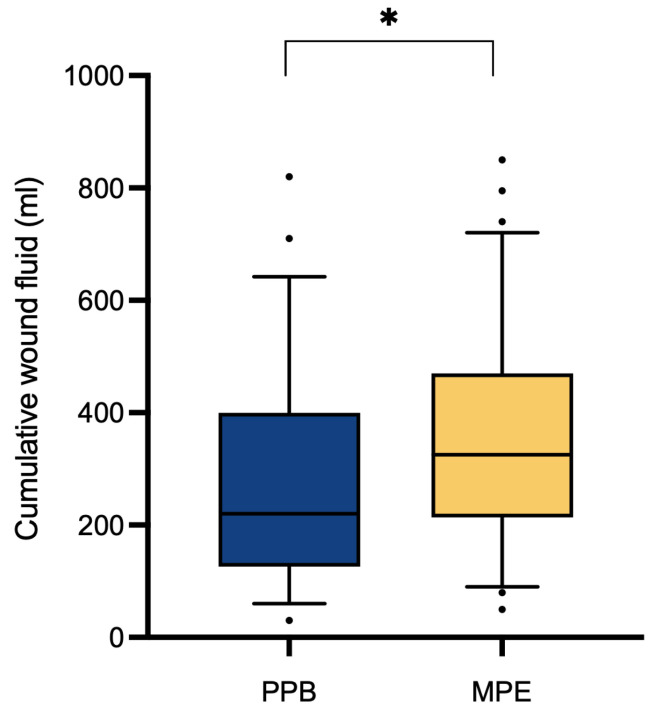
Cumulative postoperative wound fluid quantity (mL). Comparative analysis through Student’s *t*-test indicates a significant increase (p* = 0.0324) in the MPE cohort (*n* = 72) compared to the PPB cohort (*n* = 56).

Assessment of clinical outcomes through a comparative analysis between the two patient cohorts regarding the overall abdominal wound fluid production revealed a significantly (p* = 0.0324) higher quantity within the monopolar electrocautery (MPE) group (351.11 ± 185.96 mL) in contrast to the PEAK PlasmaBlade (PPB) group (279.38 ± 183.38 mL) (Table 2 and Figure 2). An analysis of hospitalization duration (10.86 ± 2.29 days vs. 11.13 ± 3.00 days, *p*^ns^ = 0.5828) and drain dwell time (5.63 ± 1.54 days vs. 5.53 vs. 1.73 days, *p*^ns^ = 0.6133) demonstrated no significant discrepancies between the two patient cohorts.

### 3.1. Risk Factors

**Figure 3:**
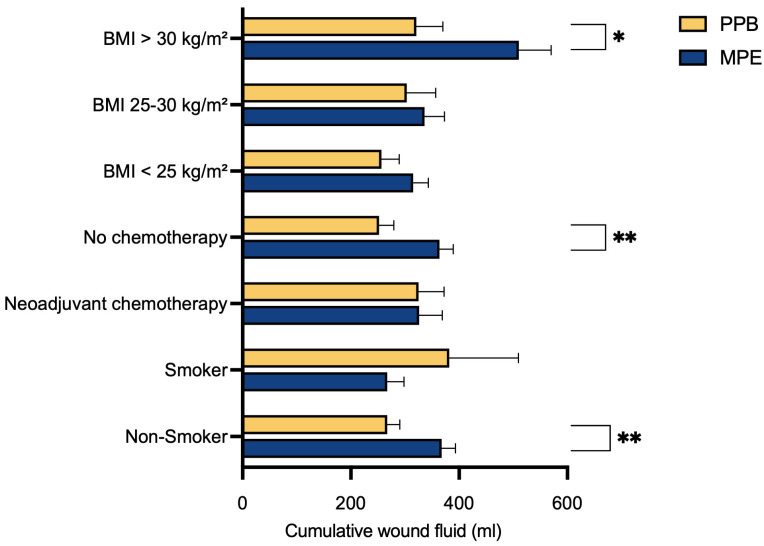
Evaluation of cumulative wound fluid quantity and risk factors. Mean and SEM of postoperative drainage volume (mL) are shown for both cohorts. Statistical significance was determined by Student’s *t*-test, revealing a significant reduction in postoperative wound fluid production after surgery with the PPB for those with a BMI exceeding 30 kg/m^2^ (p* = 0.0284), those without prior neoadjuvant chemotherapy (p** = 0.0041) and for non-smokers (p** = 0.0046). The level for statistical significance was set at * *p* < 0.05, ** *p* < 0.02.

To evaluate the potential impact of various risk factors, patients were subdivided into specific groups based on their individual characteristics, followed by a comparison. Notably, a significant reduction in postoperative abdominal wound fluid production after surgery with the PPB was observed for non-smokers (p** = 0.0046), those without prior neoadjuvant chemotherapy (p** = 0.0041) and those with a BMI exceeding 30 kg/m^2^ (p* = 0.0284) (Table 2 and Figure 3).

**Figure 4:**
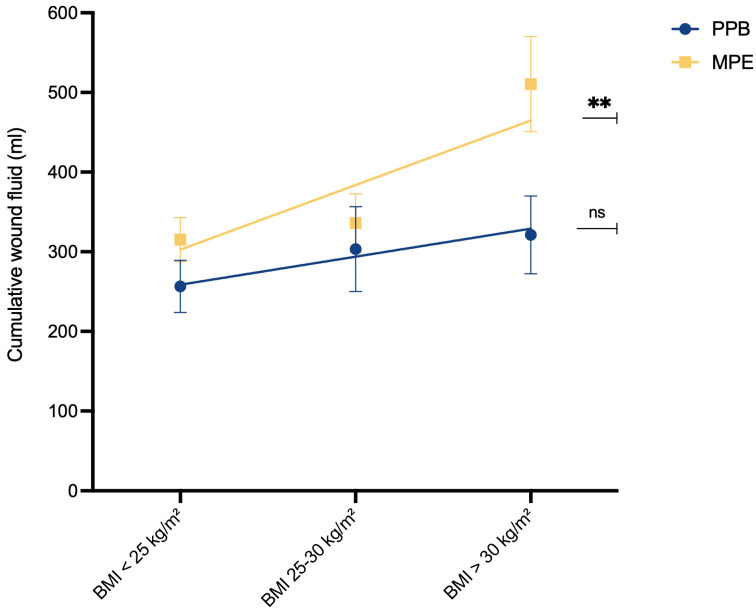
Relationship between BMI and wound fluid quantity (mL). In both groups, we compared the correlation of cumulative wound fluid quantity (mL) and BMI class by simple linear regression analysis. The analysis revealed a significant correlation between higher BMI class and increased postoperative wound fluid production in the MPE cohort. Slope significantly non-zero [PPB]: F(1.54) = 1.144, *p* ^ns^ = 0.2895, y= 35.19x + 223.4; [MPE]: F(1.70) = 8.104, p** = 0.0058, y = 81.06x + 221.6. The level for statistical significance was set at ^ns^ *p* ≥ 0.05, ** *p* < 0.02.

To assess the relationship between BMI and wound fluid production, simple linear regression analysis was conducted (Figure 4). In the MPE cohort, a significant association emerged between higher BMI and increased postoperative wound fluid production (p** = 0.0058). However, such a correlation was not observed within the PPB cohort (*p* ^ns^ = 0.2895).

Within all subgroups that exhibited favorable outcomes associated with the use of PEAK PlasmaBlade (PPB) in the context of the volume of postoperative drainage fluid, an examination of both drain dwell time and the duration of hospitalization was performed. Consistent with the observed reduction in wound fluid volume, a tendency towards decreased drain dwell time and shortened hospital stays was observed, although these trends did not achieve statistical significance (Table 3).

In the cohort of patients undergoing unilateral reconstruction, a noteworthy decrease in postoperative drainage fluid is observed in the PEAK PlasmaBlade group compared to monopolar electrocautery (p** = 0.0077). Conversely, there is no statistically significant disparity between the two devices in bilateral reconstruction cases (p^ns^ = 0.9404). Additionally, when comparing unilateral and bilateral reconstructions irrespective of the surgical device, there is no noticeable difference in postoperative wound fluid quantity (p^ns^ = 0.7118) (Table 4).

### 3.2. Postoperative Complications and Pain

An analysis of postoperative complications did not yield any statistically significant distinctions between the two patient cohorts, both in terms of overall complications (p^ns^ = 0.2758) and in the specific assessment of bleeding or hematoma (p^ns^ = 0.7128) (Table 5). All postoperative complications, as per the Clavien–Dindo [23] classification, are outlined in Table 5.

**Figure 5:**
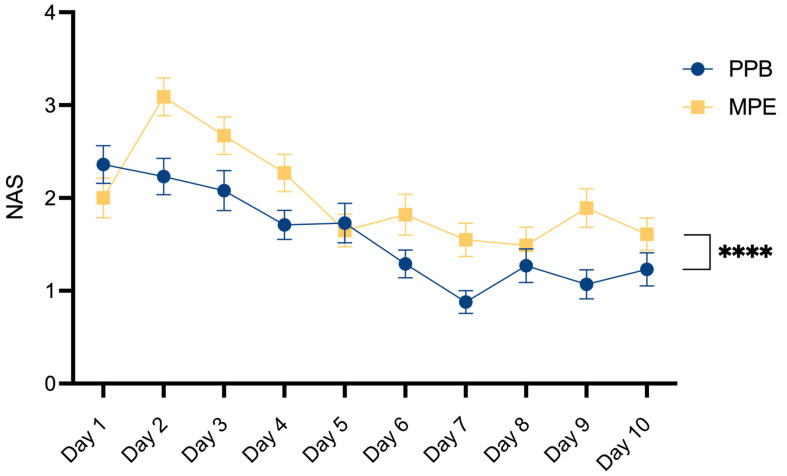
Comparative evaluation of postoperative pain. Mean and SEM for Numeric Analog Scale (NAS) assessment over a 10-day postoperative period. Statistical significance was determined by 2-way ANOVA: p**** < 0.0001, demonstrating a notable reduction in postoperative pain within the PEAK PlasmaBlade (PPB) group.

Postoperative pain assessment was conducted using a Numerical Analog Scale, graded on a scale ranging from 0 (indicating no pain) to 10 (reflecting the most severe imaginable pain). As outlined in Table 6 and Figure 5, a comparative investigation between the two patient cohorts unveiled a statistically significant decrease in postoperative pain within the PPB group (p**** < 0.0001).

As depicted in Figure 5, postoperative pain rates decreased within both studied cohorts throughout the 10-day postoperative period. We compared postoperative pain levels on day 1 versus day 10 for both patient cohorts using a Student’s *t*-test. This analysis revealed a significant decrease in pain levels in the PPB cohort (p** = 0.0067), indicating a notable reduction in pain over time. However, in the MPE cohort, while there was a decrease in postoperative pain levels between day 1 and day 10, the *t*-test did not show significance (p^ns^ = 0.4071).

## 4. Discussion

Autologous DIEP-based breast reconstruction involves extensive undermining at the abdominal donor site, resulting in sizable wound areas. Typically, flap dissection is undertaken with electrosurgical devices to allow simultaneous hemostasis and efficient operating times [24]. We evaluated two specific dissection devices—the standard monopolar electrocautery and the newer PEAK PlasmaBlade—in the context of abdominal flap harvesting, aiming to discern their impacts on clinical outcomes.

Previous research has yielded conflicting and inconclusive results regarding whether the selection of the dissection device can genuinely lead to better clinical outcomes.

Studies assessing outcomes in extensive wound areas, like those involved in autologous breast reconstruction using the abdominal donor site, have often been limited by small sample sizes [11,17,18,19,20,21,22,25,26]. While more extensive investigations have been undertaken in distinct surgical contexts, such as tonsillectomy [27,28,29] and surgical implant replacement [30], these findings may not be directly applicable to the specific circumstances of autologous breast reconstruction.

Our retrospective analysis sought to broaden the scope of this research by including a total of 128 patients in our study. Of these patients, 72 underwent abdominal flap preparation using conventional electrocautery, while 56 patients underwent the procedure with the PEAK PlasmaBlade. A comparison of these two patient cohorts revealed no statistically significant differences in age, body mass index, smoking status, history of neoadjuvant oncologic therapy, diabetes status and the primary indication for mastectomy. This comparability ensures the reliability and robustness of our observed outcomes.

Our analysis yielded the significant (p* = 0.0324) finding of a higher cumulative wound fluid quantity in the monopolar electrocautery group (351.11 ± 185.96 mL) compared to the PEAK PlasmaBlade group (279.38 ± 183.38 mL). While previous research on this topic has provided inconclusive data, some studies support our finding by reporting lower total drain output following the use of the PEAK PlasmaBlade [11,17,18,19,20,22], whereas other authors found no difference between the two dissection devices [21,25,26]. It is worth noting that, to the best of our knowledge, no prior study has reported an increase in seroma rates after utilizing the PEAK PlasmaBlade. It has previously been suggested that the reduced working temperature of the PEAK PlasmaBlade leads to lower tissue damage [8,10], which contributes to reduced seroma formation. One study in gender-affirming mastectomy patients has investigated histologic samples, showing a 22% reduction in thermal injury depth with the PlasmaBlade compared to conventional monopolar cautery [18].

Similarly, the existing literature on the evaluation of hospitalization and drain dwell time yields inconsistent results. While Schlosshauer, Dogan and Sowa reported positive outcomes in the PlasmaBlade cohorts [11,17,19,20], other studies did not identify variations in terms of hospitalization and drain dwell time [21,25,26]. Our data did not reveal a difference between both devices for these aspects. There is no previous work indicating inferiority of the PlasmaBlade in this regard.

We intentionally selected abdominal-based autologous breast reconstruction as our research focus. This patient group not only represents a highly standardized approach to large-scale wound preparation but also encompasses a spectrum of patient-specific risk factors, including those related to oncologic treatments, which are pertinent to our analysis. Extensive cohort studies conducted so far have failed to establish a definitive link between neoadjuvant chemotherapy and the occurrence of surgical complications during breast reconstruction [31,32,33,34,35]. Nevertheless, we were determined to assess the role of dissection instruments in relation to neoadjuvant chemotherapy and other risk factors and its impact on surgical outcomes. We identified chemotherapy, smoking status and BMI as the primary risk factors of interest in our patient cohorts, with prevalences high enough to enable statistical analysis.

In our multivariate data analysis, we identified a substantial reduction in postoperative abdominal wound fluid production in the PEAK PlasmaBlade cohort compared to the electrocautery cohort across three specific subgroups. These groups comprised individuals without prior neoadjuvant chemotherapy (p** = 0.0041), non-smokers (p** = 0.0046) and those with a BMI exceeding 30 kg/m^2^ (p* = 0.0284).

It is noteworthy that these groups typically do not share the same risk profile. In standard practice, non-smokers and patients without previous neoadjuvant chemotherapy are typically regarded as low-risk individuals for surgical complications. Conversely, patients with a BMI exceeding 30 kg/m^2^ are commonly perceived as a high-risk population for adverse events in the surgical context [36]. Mani et al. previously investigated the association between BMI and the occurrence of donor-site seroma following the harvesting of DIEP flaps. Their findings revealed that obese patients (BMI > 30 kg/m^2^) had the highest incidence of postoperative seroma formation, reaching a rate of 16% [16]. Our results revealed a significant association between higher BMI and increased postoperative wound fluid production (p** = 0.0058) within the MPE cohort (Figure 4). In contrast, no such correlation was evident within the PPB cohort (p^ns^ = 0.2895), suggesting that the utilization of PPB might mitigate the risks associated with higher BMI.

Although not statistically significant, all subgroups that displayed positive outcomes with the use of the PEAK PlasmaBlade regarding postoperative drainage volume also demonstrated a trend toward reduced drain dwell time and shorter hospital stays.

The assessment of hospitalization duration may be subject to some inaccuracies, as our department typically retains patients in the hospital until histopathological results become accessible, and the removal of the monitoring skin island is performed. Nevertheless, it is noteworthy that a tendency towards reduced drainage catheter dwell time in the PPB group may potentially be linked to our observed outcomes of a significant decrease in postoperative pain levels around day 7 and day 9 in the PPB group, attributable to the earlier removal of drainage devices.

Our data reveal a significant decrease in postoperative pain within the PEAK PlasmaBlade cohort. To our knowledge, only Friebel et al. have previously compared postoperative pain levels between both dissection devices, reporting an increase in the PPB cohort [21]. This was attributed to the potential impact of tighter abdominal closure due to lower flap weights in this group. In our patient cohorts, both flap volume (*p* = 0.8572) and the distribution of unilateral versus bilateral reconstruction (*p* = 0.0560) within the two patient groups were comparable, as an analysis of our patient characteristics (Table 1) showed. We consider this as a strength of our study since it allows the assumption that postoperative pain levels are not biased by these characteristics. There have been studies examining postoperative pain following surgical skin incisions made with electrocautery versus steel scalpel, which reported reduced pain levels and a decreased use of analgesics in the electrocautery group [37,38]. Chrysos explains this by highlighting that the vaporization of cells resulting from the application of pure sinusoidal current leads to immediate tissue and nerve necrosis without significantly affecting nearby structures [38]. The varying degrees of nerve damage between electrocautery and the PEAK PlasmaBlade may also contribute to our significant finding of reduced postoperative pain in the PPB group, but such evaluations should be a focus of future studies.

Our study demonstrates also several limitations. Due to retrospective data analysis, we cannot offer detailed information concerning the adjustments of the used monopolar electrocautery. But we tried to overcome this lack of information by only including patients that were operated under the lead of one single surgeon, so it may be assumed that the same preferred settings were used. Furthermore, this study benefits from the extensive experience of this single surgeon, contributing to the reliability of the findings and minimizing the risk of performance bias. Moreover, the similarity in patient demographic characteristics across both cohorts indicates a low risk of selection bias, despite the study’s retrospective nature.

## 5. Conclusions

In conclusion, our findings align with previous research, indicating that the PEAK PlasmaBlade is not inferior to conventional electrocautery. Furthermore, patients may experience advantages such as reduced drainage volume and lower postoperative pain levels following wound preparation with the PlasmaBlade. Notably, our analysis identified patient subgroups that could derive even greater benefits from the use of this device. In these specific patient groups, utilizing the PEAK PlasmaBlade could result in better clinical outcomes. Therefore, the choice to use this device may be based on the potential benefits for patients, rather than solely on the surgeon’s subjective preferences.

## Figures and Tables

**Table 1:** Patient characteristics.

	PPB	MPE	
*n*	56	72	
	Mean (±std)	Mean (±std)	*p*-Value
Age	49.68 (±10.50)	48.67 (±9.56)	0.5733
BMI	25.37 (±3.85)	25.33 (±4.01)	0.9612
Flap volume (mL)	617.16 (±283.66)	608.27 (±322.90)	0.8572
	***n* (%)**	***n*%**	***p*-value**
Female	56 (100)	72 (100)	>0.999
Indication			
Carcinoma	53 (94.64)	71 (98.61)	0.2005
Prophylactic	3 (5.36)	1 (1.39)	
Breast reconstruction			
Unilateral	34 (60.7)	55 (76.4)	0.0560
Bilateral	22 (39.3)	17 (23.6)	
Nicotine			
Yes	6 (10.71)	12 (16.67)	0.3366
No	50 (89.29)	60 (83.33)	
Diabetes			
Yes	1 (1.79)	0 (0.00)	0.3918
No	55 (98.21)	72 (100.00)	
Neoadjuvant chemotherapy			
Yes	21 (37.50)	24 (33.33)	0.6243
No	35 (62.50)	48 (66.67)	
BMI			0.9692
BMI < 25 kg/m^2^	32 (57.14)	40 (55.55)	
BMI 25–30 kg/m^2^	15 (26.79)	21 (29.17)	
BMI > 30 kg/m^2^	9 (16.07)	11 (15.28)	

**Table 2:** Clinical outcome.

	PPB	MPE	
Outcome	Mean (±std)	Mean (±std)	*p*-Value
Number of suction drainages	2.00 (±0.00)	1.99 (±0.12)	0.3799
Duration of draining (days)	6.18 (±1.69)	6.29 (±1.72)	0.7128
Hospitalization (days)	10.86 (±2.29)	11.13 (±3.00)	0.5828
Total wound fluid quantity (mL)	279.38 (±183.38)	351.11 (±185.96)	0.0324 *
**Seroma (mL) and risk factors**			
Non-Smoker	267.10 (±164.21)	367.92 (±195.73)	0.0046 **
Smoker	381.67 (±313.52)	267.08 (±107.84)	0.2612
Adjuvant Chemotherapy	325.00 (±215.56)	326.04 (±209.19)	0.9870
No Chemotherapy	252.00 (±161.23)	363.65 (±176.29)	0.0041 **
BMI < 25 kg/m^2^	256.41 (±186.32)	315.25 (±175.01)	0.1727
BMI 25–30 kg/m^2^	303.33 (±206.08)	335.95 (±168.64)	0.6053
BMI > 30 kg/m^2^	321.11 (±146.33)	510.45 (±197.74)	0.0284 *

The level for statistical significance was set at * *p* < 0.05, ** *p* < 0.02.

**Table 3:** Evaluation of drain dwell time and hospitalization within subgroups.

	PPB	MPE	
Drain Dwelling Time (d)	Mean (±std)	Mean (±std)	*p*-Value
Non-Smoker	6.10 (±1.50)	6.35 (±1.71)	0.4210
No chemotherapy	6.09 (±1.50)	6.35 (±1.62)	0.4440
BMI > 30 kg/m^2^	6.67 (±1.87)	7.55 (±1.75)	0.2933
**Hospitalization (d)**			
Non-Smoker	10.86 (±2.35)	11.00 (±2.26)	0.7513
No chemotherapy	10.94 (±2.71)	11.02 (±3.22)	0.9077
BMI > 30 kg/m^2^	11.44 (±4.03)	11.73 (±1.19)	0.8267

**Table 4:** Evaluation of cumulative abdominal wound fluid quantity (mL) in uni- and bilateral breast reconstruction.

	PPB	MPE	
	Mean (±std)	Mean (±std)	*p*-Value
Unilateral	248.53 (±177.81)	357.09 (±184.89)	0.0077 **
Bilateral	327.05 (±189.92)	331.76 (±199.31)	0.9404
	**Unilateral reconstruction**	**Bilateral reconstruction**	
	**mean (±std)**	**mean (±std)**	***p*-value**
All patients (*n* = 128)	315.62 (±188.80)	329.10 (±191.48)	0.7118

The level for statistical significance was set at ** *p* < 0.02.

**Table 5:** Postoperative complications.

	PPB	MPE	
	*n* (%)	*n*%	*p*-Value
Complications	13 (23.21)	23 (31.94)	0.2758
Bleeding/Hematoma	4 (7.14)	4 (5.56)	0.7128
**Clavien–Dindo**			
1	5 (8.93)	10 (13.89)	
Bleeding/Hematoma	2 (3.57)	1 (1.39)	
Wound healing complications	3 (5.36)	7 (9.72)	
Seroma	0 (0.00)	2 (2.78)	
2	0 (0.00)	0 (0.00)	
3a	0 (0.00)	2 (2.78)	
Wound healing complications	0 (0.00)	2 (2.78)	
3b	8 (14.29)	11 (15.28)	
Bleeding/Hematoma	2 (3.57)	3 (4.17)	
Wound healing complications	6 (10.71)	8 (11.11)	

**Table 6:** Postoperative pain, Numerical Analog Scale (NAS) (0–10).

	PPB	MPE	
	Mean (±std)	Mean (±std)	*p*-Value
Day 1	2.36 (±1.52)	2.00 (±1.81)	0.4994
Day 2	2.23 (±1.47)	3.09 (±1.73)	0.0080 **
Day 3	2.08 (±1.62)	2.67 (±1.71)	0.0683
Day 4	1.71 (±1.18)	2.27 (±1.70)	0.0512
Day 5	1.73 (±1.59)	1.65 (±1.50)	0.7855
Day 6	1.29 (±1.12)	1.82 (±1.87)	0.0729
Day 7	0.88 (±0.93)	1.55 (±1.52)	0.0065 **
Day 8	1.27 (±1.35)	1.49 (±1.66)	0.4455
Day 9	1.07 (±1.18)	1.89 (±1.78)	0.0153 **
Day 10	1.23 (±1.34)	1.61 (±1.48)	0.2928

The level for statistical significance was set at ** *p* < 0.02.

## Data Availability

The data presented in this study are available on request from the corresponding author.
